# The Role of Neutrophils as a Driver in Hepatic Ischemia-Reperfusion Injury and Cancer Growth

**DOI:** 10.3389/fimmu.2022.887565

**Published:** 2022-07-01

**Authors:** Christof Kaltenmeier, Hamza O. Yazdani, Sanah Handu, Brandon Popp, David Geller, Samer Tohme

**Affiliations:** ^1^ Department of Surgery, University of Pittsburgh Medical Center, Pittsburgh, PA, United States; ^2^ Lake Erie College of Osteopathic Medicine, Erie, PA, United States

**Keywords:** neutrophil, ischemia reperfusion (I/R) injury, neutrophil extracelluar traps, cancer liver, liver transplantation

## Abstract

The innate immune system plays an essential role in the response to sterile inflammation and its association with liver ischemia and reperfusion injury (IRI). Liver IRI often manifests during times of surgical stress such as cancer surgery or liver transplantation. Following the initiation of liver IRI, stressed hepatocytes release damage-associated molecular patterns (DAMPs) which promote the infiltration of innate immune cells which then initiate an inflammatory cascade and cytokine storm. Upon reperfusion, neutrophils are among the first cells that infiltrate the liver. Within the liver, neutrophils play an important role in fueling tissue damage and tumor progression by promoting the metastatic cascade through the formation of Neutrophil Extracellular Traps (NETs). NETs are composed of web-like DNA structures containing proteins that are released in response to inflammatory stimuli in the environment. Additionally, NETs can aid in mediating liver IRI, promoting tumor progression, and most recently, in mediating early graft rejection in liver transplantation. In this review we aim to summarize the current knowledge of innate immune cells, with a focus on neutrophils, and their role in mediating IRI in mouse and human diseases, including cancer and transplantation. Moreover, we will investigate the interaction of Neutrophils with varying subtypes of other cells. Furthermore, we will discuss the role and different treatment modalities in targeting Neutrophils and NETs to prevent IRI.

## Introduction

Liver ischemia and reperfusion injury (IRI) often presents in various surgical procedures such as cancer surgery or liver transplantation (1, 2). Orthotopic liver transplantation (OLT) is the current standard of care for patients with end-stage liver disease and selected hepatic malignancies. Early graft failure is common with an incidence of approximately 25% and a major contributor to morbidity and mortality (3). IRI is one of the leading causes of early graft dysfunction and a major factor for acute and chronic rejection (4). IRI also plays an important role in the setting of liver cancer surgery (2). Here, studies have shown that prolonged IRI promotes the growth of micro metastatic disease within the liver from primary or metastatic cancer (5). Prolonged IRI is strongly associated with early disease recurrence and decreased survival.

Prolonged IRI leads to a lack of oxygen and nutrient supply for cells within the liver parenchyma. During this time hepatocytes and Kupffer cells undergo anaerobic metabolism, reactive oxygen production, and subsequent cell death along with the release of damage associated patterns (DAMPs). Upon reperfusion, DAMPs are flushed into the circulation and a complex cascade of inflammatory mediators that promote tissue damage is initiated.

Although the reperfusion phase of the liver is the principal driver of tissue injury, the pretransplant cold storage itself can trigger organ damage (1, 6, 7). Studies have shown that several apoptosis inducing kinases are activated in parenchymal liver cells following cold-induced tissue injury, which subsequently promote cell death upon reperfusion 1. Reducing the cold ischemia time and limiting the damage of reperfusion are important targets in order to reduce graft dysfunction and rejection.

Liver damage is mostly caused during reperfusion when host cells are in metabolic anaerobic distress and excess infiltration of innate immune cells occurs. Neutrophils are among the first cells to arrive within the liver following IRI (5, 8, 9). There is overwhelming evidence that excessive neutrophil infiltration contributes to the pathogenesis of IRI. Neutrophil-induced liver injury is a multistep process that starts with neutrophil activation, recruitment of these cells to site of injury *via* transmigration, interaction with hepatic host and other immune cells (10).

**Figure 1:**
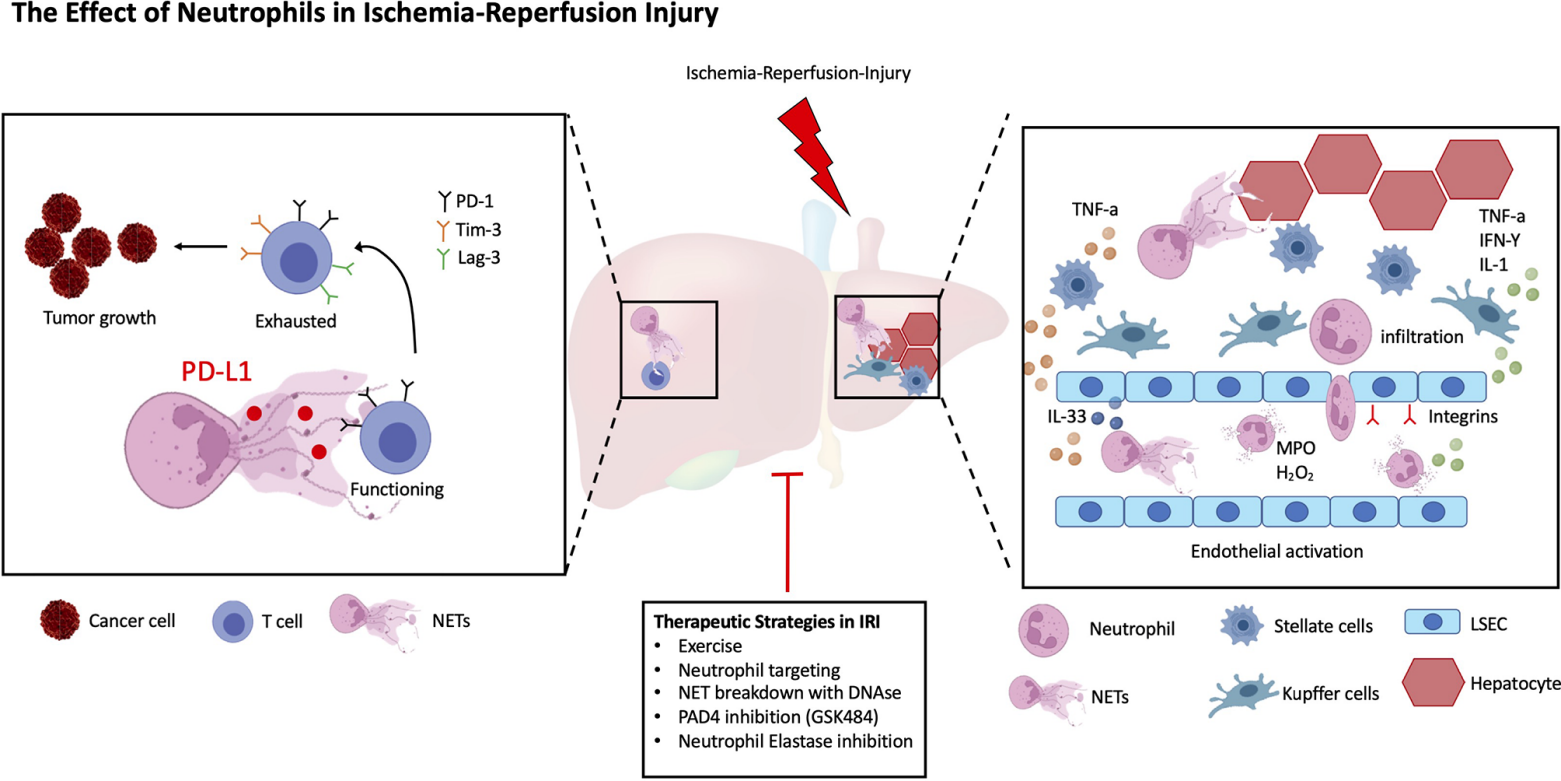
The Role of Neutrophils in Ischemia-Reperfusion Injury. A simplified schematic representation of the roles of Neutrophils in liver IRI. Following IRI liver parenchymal cells promote the recruitment, activation, and phenotypic differentiation of neutrophils. LSECs increase the production of NETs *via* IL-33 signaling, in addition they upregulate adhesion markers to facilitate Neutrophil transmigration, extravasation and NET formation. Similarly, both HSCs and Kupffer cells secrete cytokines to increase Neutrophil homing. Within the tumor microenvironment (TME), NETs promote the exhaustion of T cells leading to increased tumor burden. Several, both non-pharmacological and pharmacological therapies have been described to decrease liver IRI.

In this review, we will discuss the current advances in understanding the role of neutrophils in cold and warm liver IRI, with a specific focus on the role and function of activated neutrophils and their interaction with liver parenchymal host and infiltrating immune cells. We will furthermore discuss non-pharmacological and pharmacological treatment options to limit IRI and/or decrease NET formation in the liver (**Figure 1**).

## The Role of Liver Resident Cells in Liver I/R and Their Role in Neutrophil Recruitment

IRI results in tissue and cellular necrosis which are the main contributors to liver damage following transplantation or cancer surgery (11). Even in the absence of microbial pathogens, excessive activation of a sterile inflammatory response following the restoration of blood flow can result in further dysfunction. The signaling events contributing to local hepatocellular damage are diverse and complex, and involve the interaction between hepatocytes, liver sinusoidal endothelial cells (LSEC), Kupffer cells (KC), and hepatic stellate cells (HSC), all leading to neutrophil activation and formation of neutrophil extracellular traps (NETs) (10, 12). During organ procurement and transplantation, the liver undergoes two insults classified as cold storage and warm IRI. During cold storage, organs are deprived of blood flow and placed on ice to preserve their function and limit cellular metabolism. This is followed by warm IRI upon reperfusion of the liver. During cold and warm IRI, LSECs undergo varying alterations including nuclear membrane vacuolization and structural shifting towards a rounded shape. In addition, LSEC exhibit marked increased levels of reactive oxygen species (ROS) that decrease LSECs viability (12). The role of LSECs in liver injury has been reported in many experimental studies including: 1. Upregulation of surface adhesion molecules 2. Deregulation and disassembly of cell structure due to an increment in calcium-derived calpain activity 3. Apoptosis of LSEC to elastase-mediated paracrine interactions with leucocytes (13, 14).

Our group has previously reported that in a murine surgical model of *in situ* warm IRI (1h ischemia followed by 6h reperfusion time), increased activation of endothelial cell prompts the release of Interleukin 33 (IL-33) resulting in increased neutrophil cell activation and NET formation. The response to IL-33 signaling is determined by the expression of its surface receptor ST2 on the target cell which in humans are located on chromosome 11p14.3-p12. We were among the first group to show that mouse bone marrow-derived neutrophils express ST2 and that its expression is markedly increased by IL-33 stimulation. In addition, human data reveals that increased IL-33 serum levels are strongly associated with post-operative NET formation in individuals undergoing liver resection for hepatic malignancies (15).

In tandem with LSEC, activated Kupffer cells (KCs), resident macrophages in the liver, play an important role in the homeostatic response to IRI. Jaeschke et al. have shown that rat KCs when treated with high dose of retinol or propionibacterium acnes-activated KCs significantly upregulated plasma glutathione disulfide and ROS production (GSSG, an index for oxidative stress), while treatment with methyl palmitate or gadolinium chloride lead to formation of inactivated KCs leading to significant decrease in plasma GSSG levels. Inactivated KCs significantly protected the liver from IRI (16). Similarly, a study published by Dai et al. showed significant increase in the production and release of ROS and proinflammatory cytokines following IRI (17). Of the cytokines released by KCs, TNF-α has been shown to induce intraluminal expression of adhesion molecules such as ICAM-1 and P-selectin that facilitates adhesion of circulating neutrophils through a rolling and binding motion and further facilitates cell extravasation (18, 19). Since activated KCs also play a role in neutrophil chemotaxis, both TNF-α and IL-1 that are released from KCs upregulate MAC-1 adhesion protein on the neutrophil surface and promote the synthesis of IL-8 (20). In addition, Su et al. reported that KCs derived TNF-α promotes hepatocyte chemokine CXCL1 induction through the NF-κB pathway to mobilize neutrophils towards injured areas (21).

HSCs have been well studied in their role of IR. HSCs are quiescent, however during liver injury they release retinoids and can undergo differentiation into a myofibroblast-like phenotype. This activation is associated with loss of GFAP and the expression of alpha-smooth muscle actin (α-sma). A recent publication by Hwang et al. has shown that activated HSCs promote liver fibrosis *via* transfer of retinol form HSCs to hepatocytes *via* STRA6 (Stimulated by retinoic acid 6). This finding was confirmed in the liver of cirrhotic patients showing high expression of STRA6 compared to normal liver controls (22). While HSCs have been noted to directly communicate with immune cells inside sinusoids following endothelial cell damage, there is still much to investigate regarding their capacity in their ability to recruit immune cells along with regulation of sinusoidal inflammation, especially in the context of I/R injury (23). Puche et al. developed a model for depleting HSCs to investigate its properties in liver I/R injury. They utilized transgenic mice that expressed herpes simplex virus Thymidine Kinase gene (HSV-Tk) and treated them with carbon tetrachloride for 10 days to promote proliferation and subsequent making them susceptible to cell killing by treating them with ganciclovir. Approximately, 70% of HSCs were depleted and intrahepatic neutrophil infiltration was significantly decreased. Furthermore, they reported that expression of TNF-α, CXCL1, and endothelin-A receptor were also attenuated. According to these findings, HSCs are implicated in hepatic CXCL1 synthesis and contribute to neutrophil recruitment and microcirculatory failure caused by endothelin-A receptor stimulation (24, 25).

## The Role of Neutrophils in I/R Induced Inflammation

Neutrophils, an essential component of IRI, are one of the first cell types to be recruited from the bloodstream to sites of injury. Neutrophils are recruited to ischemic sites *via* DAMPs, which are produced by intracellular organelles and the extracellular matrix of damaged cells (26). DAMPs, such as HMGB1, S100 proteins, heat shock proteins, circulating RNA/DNA all trigger a broad variety of inflammatory responses that promote the secretion of pro-inflammatory cytokines and chemokines. These circulating mediators promote the upregulation of adhesion molecules on the endothelium, acute inflammation and the recruitment of neutrophils to the site of injury (27). Within the liver, Neutrophil induced inflammation is further maintained *via* CXC chemokines, generated by hepatocytes. Within the ischemic tissue, neutrophils form hydrogen peroxide and myeloperoxidase (MPO) (28). Hydrogen peroxide induces intracellular oxidant stress by direct diffusion into hepatocytes. MPO uses hydrogen peroxide to create hypochlorous acid, which enters target cells and causes damage. Neutrophil elastase secreted by activated neutrophils has been shown to inhibit PGI2 production. PGI2 is a vasodilator that is released from endothelial cells and is protective during hepatic IRI by maintaining proper hepatic circulation. When activated neutrophils release neutrophil elastase, the protective effect of PGI2 is inhibited, and hepatic injury ensues (29, 30).

Complement induced recruitment of neutrophils plays an important role in liver IRI. The complement system is part of the innate immune system and can be activated by one of three pathways. These pathways include the classical pathway, which is antibody dependent, the alternative pathway and the mannose-binding lectin pathway. In IRI, the complement cascade is activated by detecting cellular contents that were released into the extracellular space as a result of ischemia. Complement activation leads to the formation of the soluble bioactive peptides, C3a and C5a, and the membrane attack complex, which results in the recruitment of inflammatory cells. C5a, from the complement system, stimulates Kupffer cell activation, which produces TNF-alpha, IL-1 and ROS. This ultimately leads to neutrophil recruitment and influx into liver sinusoids to promote further inflammation (31).

Another major contributing factor of neutrophil-induced inflammation is the formation of neutrophil extracellular traps (NETs). NETs are net-like structures, composed of a meshwork of chromatin fibers that contain enzymes such as MPO, neutrophil elastase, and cathepsin G. NET release from neutrophils is believed to happen *via* two different mechanisms. The first method, termed NETosis, is through the release of chromatin and granular contents into the extracellular space as the plasma membrane dissolves. Although similar to apoptosis, neutrophils appear to not be cleared by phagocytic cells after undergoing NETosis because they do not display apoptotic signals and therefore remain alive. The second method of NET release is *via* DNA/serine protease released from intact neutrophils (32). NETs have also been shown to activate the complement system and thereby further contribute to inflammation. NETs stimulate components necessary for the formation of C3bBb, which is eventually necessary for membrane attack complex formation (33).

*In vitro* studies have shown that Neutrophils increase the death of hepatocytes and KCs and thereby promote the release of more proinflammatory cytokines and DAMPs such as HMGB1 and histones. These DAMPs then further promote the stimulation of NETs through Toll-like receptor (TLR4)- and TLR9-MyD88 signaling pathways (34, 35).

Neutrophils tend to have relatively short lifespans, surviving for less than 24 hours in the bloodstream before undergoing apoptosis and clearance by macrophages. During periods of inflammation, numerous factors have been shown to inhibit neutrophil apoptosis and prolong their survival. It has been shown that hepatic IRI upregulates OX40, a costimulatory molecule that is constitutively expressed in human peripheral neutrophils. Increased OX40 levels promote neutrophil survival, prolonging the neutrophil release of proinflammatory factor (36). Myeloid cell leukemia-1 (MCL-1), an anti-apoptotic member of the Bcl-2 family, has been shown to downregulate neutrophil apoptosis, and promote its survival. MCL-1 has been shown to be upregulated in in models of cerebral IRI, which promote neutrophil survival (37). Other factors including DAMPs, lipid mediators, hormones, chemokines, and cytokines have been described as neutrophil survival factors (38, 39).

NETs play an important role in hepatic IRI in the context of acute rejection. Liu et al. have shown in their study that HMGB1 upon liver transplantation contributes to the development of acute liver rejection. It has been very well characterized that HMGB1 through TLR4 and RAGE receptor can activate neutrophils and promote NET formation. The authors observed increased NET levels in the serum of transplanted patients which negatively corelated with immediate postoperative liver function. In addition, both HMGB1 and NET levels correlated positive in the serum following transplantation. They further show that hepatic Kupffer cells where the main source of HMGB1 production and were polarized to M1 phenotype through NETs. Furthermore, a combination of TAK-242 (a TLR-4 inhibitor) as well as rapamycin reduced the damaging effects of acute rejection after liver transplantation more efficiently than either of them alone (40).

## Neutrophil Extracellular Trap and Its Interaction With Platelets

Accumulating evidence shows that patients that underwent liver surgery are hypercoagulable in the immediate post-operative setting (41). The mechanism of this pro-coagulation state in the postoperative period is still under investigation. We have previously shown within a murine model of liver IRI, induction of NETs can activate platelets resulting in systemic immune-thrombosis and distant organ injury (42, 43). Our data showed that 1-hour of ischemia followed by 6 hours of reperfusion significantly activated platelet and increased platelet-neutrophil aggregation. This led to increased NET-platelet rich micro-thrombi in the microvasculature after liver IRI. When NETs were blocked by DNase-1 therapy, immunological thrombi and organ damage were significantly reduced. In addition, we observed a significant decrease in the NET induced platelet activation in a TLR4 depleted platelets. Furthermore, when compared to control mice, platelet-specific TLR4 KO animals showed much less distant organ damage, with lower circulating platelet activation and platelet-neutrophil aggregates following liver I/R.

## Neutrophils Crosstalk With Adaptive Immune Cells In the Tumor Microenvironment

Recent studies have shown that Neutrophils have bidirectional interactions with B- and T-lymphocytes subsets. *In vitro*, human Neutrophils have been shown to secrete cytokines that are crucial in the survival and maturation of B-lymphocytes. These cytokines include B‐cell‐activating factor of the tumor necrosis factor family (BAFF) and A Proliferation‐Inducing Ligand (APRIL). These findings have been substantiated by the discovery of pro-helper Neutrophils in the perifollicular area of the spleen in humans, these Neutrophils were therefore named B-cell helper neutrophils. These Neutrophils secrete higher amounts B cell stimulating and attracting factors such as CD40L, interleukin-21 and CXCL12. Interestingly, human splenic Neutrophils have been shown to lose their selective perifollicular topography and start to infiltrate the germinal centers of splenic follicles under systemic inflammatory stress (44). Tumor associated Neutrophils (TANs) have been shown to promote B cell chemotaxis to the tumor by secretion of TNFα thereby playing a role in tumor progression (45).

During liver IRI, Neutrophils can stimulate the recruitment and activation of CD8+ T cells through varying cytokines and chemokines expressed within the chromatin of activated Neutrophils (46). In addition, Neutrophils can serve as antigen-presenting cells to CD4+ T cells and cross-presenting cell to CD8+ T cells (44, 47). Several studies have shown important crosstalk between neutrophils and liver-resident lymphocytes (48). Recent studies by our laboratory have shown that NETs can directly promote the exhaustion of T cells that are found within the liver following IRI. NETs harbor the immunomodulating protein program-death ligand 1 (PD-L1) which binds PD-1 on activated T cells to render them non-functioning and exhausted. This discovery represents a novel finding with possible clinical implications to overcome T cell exhaustion and tumor growth by targeting neutrophils and NETs (9, 49).

## Non-Pharmacological Methods to Target Neutrophil Mediated Liver Ischemia-Reperfusion Injury

There is broad epidemiologic and observational evidence that regular physical exercise reduces the risk of cancer, slows tumor progression, and improves outcomes when combined with other oncologic treatment strategies. A variety of trials have shown that regular exercise can improve cancer prognosis and therefore should be seen as an important adjunct to conventional treatments. Over the last decade, a lot of emphasis has been placed on preoperative rehabilitation of frail patients who have any impairment in activities of daily living. The role of pre-habilitation is to optimize functional status in frail patients undergoing major surgery. Pre-operative exercise in the non-frail patients can have several effects that are postulated to be secondary to changes in the immune system resulting in changes in immune cell subsets, infiltration of immune cells to the site of injury, as well as secretion of inflammatory mediators (50).

We have shown that 1 hour of daily exercise for 4 weeks confers sustainable protection against IRI in a murine model. Exercise-trained mice (ExT) that underwent IRI showed a decrease in liver necrosis, significantly diminished hepatic chemokine levels, and fewer infiltrating innate immune cells. Interestingly, we observed that ExT conditioned neutrophils (isolated from circulation or bone marrow) showed decreased in NET formation when stimulated with phorbol myristate acetate (PMA). We also found that exercise reduced the inflammatory cytokine storm and decreased neutrophil adhesion and migration by downregulating endothelial adhesion molecules and increased the infiltration of M2 phenotypic anti-inflammatory macrophages. Furthermore, ExT suppressed tumor metastatic growth when injected through splenic vein 4 weeks after training and enhanced NK cell infiltration to the tumor. This is of interest, since M2 phenotypic macrophages generally known to be anti-inflammatory but pro-tumorigenic in nature. In our model of preconditioning mice with ExT, the anti-inflammatory state that developed over a 4-week period lead to increased infiltration of NK cells into the liver which attenuated the growth of micrometastatic disease. We believe that the ratio of M1/M2 macrophages in the liver of ExT mice is important to determine the actual effect on the tumor, however the presence of NK and other adaptive immune cells dictates the anti-tumor immunity within the TME. Further single-cell experience will need to be carried out in ExT vs. sedentary mice to fully delineate the type of immune cells infiltrating the liver.

However, the mechanism of ExT in preventing IRI and thereby reducing the effect on cancer growth is an important and novel observation. This finding of aerobic ExT as a nonpharmacological therapy before liver surgery might provide a rationale to extend these studies to other clinical scenarios of liver IRI such as transplantation (19).

## Targeting of Neutrophil Extracellular Traps to Prevent Liver Ischemia-Reperfusion Injury

Oxidative stress is the inevitable feature of liver IRI. Excessive levels of superoxide (O2-) generated through hypoxanthine and xanthine oxidase can promotes proinflammatory milieu. Several studies, by utilizing antioxidant therapy, has shown to reduce liver IR injury in animal models (51). We have previously shown that superoxide stimulates neutrophils to release NETs through the TLR4/NOX signaling pathways. Our findings have shown that treating mice with allopurinol (superoxide inhibitor) in combination with diphenyleneiodonium (NOX inhibitor) attenuated NET formation and significantly decreased liver injury (52).

Similarly, using a mathematical model of Dynamic Network Analysis (DyNA) in a mouse model of warm *in situ* liver IRI we have previously shown that the inflammatory cytokine IL-17 plays a central role in mediating and sustaining a pro-inflammatory environment promoting I/R induced injury (53). The injury was reversed when an IL-17 neutralizing antibody was administered prior to IR injury. In addition, increase in the serum levels of IL-17 directly corresponded with the increase of intrahepatic neutrophil infiltration and NET formation suggesting IL-17 as a potent NET inducer. Similarly, in a mouse model of renal IRI, it was observed that infiltrating neutrophils were the major source of IL-17 production which further facilitated neutrophil transmigration. Furthermore, Lin et al. has shown that IL-17 positive neutrophils that are abundantly present in the human psoriasis lesions can release IL-17 through the induction of NETs. Hence targeting IL-17 using IL-17 neutralizing antibody can decrease IR induced injury *via* decreasing NET formation (54).

More recently, in a liver transplantation model, use of recombinant thrombomodulin (TM) has been shown to target NET formation and subsequently decreased liver injury in rat models. TM is a glycoprotein that is highly expressed by endothelial cells and has shown to play a protective role against liver IRI in both mouse and rat models. A study published by Yanyao et al. observed a significant decrease in neutrophil infiltration and NET formation by pretreating rats with recombinant TM (5mg/kg) intravenously 1h before the transplantation. They further show that recombinant TM suppressed TLR-4 activity and its downstream extracellular signal (kinase/c-Jun NH2 terminal kinase and NADPH/reactive oxygen species/PAD4) signaling pathways, mostly regulated by stressed released DAMPs (55).

NETs harbor a wide variety of proinflammatory cytokines and chemokines within the NET chromatin. Protein arginine deiminase 4 (PAD4) is a key mediator of NET formation. PAD4 facilitates histone citrullination to promote chromatin decondensation and the expulsion of chromosomal DNA. Studies have shown that global PAD4 knockout mice have diminished NET production and decreased primary and metastatic cancer growth compared to wild-type mice. Similar effects have been shown by targeting neutrophil elastase (NE) or using specific MPO-inhibitors in murine models. These studies were carried out by injecting a colorectal cancer cell line (MC-38) in subcutaneous or intra-splenic fashion into wildtype or PAD4 KO animals. Wildtype animals were further treated with a NE inhibitor (56-58). PAD4KO or wildtype animals treated with NE inhibitor showed significant smaller tumors with less neutrophil infiltration and NETs compared to wildtype animals.

Another important target is the destruction of NET chromatin within the tumor. DNA, the backbone of NETs can be targeted using DNase-I. In fact, DNase-I treatment has shown promising results in preclinical murine cancer models as well as clinical trials in patients with lupus nephritis, rheumatoid arthritis or cystic fibrosis. DNAse-I has a very short half-life within the circulation and therefore requires multiple injections to achieve effective concentrations. Several inhibitors of NE and PAD4 showed decreased tumor growth when tested in the tumor burdened animal models. Several pre-clinical trials in non-cancer patients using these targets have been performed, it is therefore reasonable that these inhibitors could be beneficial and improve clinical outcomes in cancer patients alike (59-62).

In addition, blocking the direct crosstalk of NETs with adjacent cancer cells in the tumor microenvironment has shown promising results in reducing the NET effect on cancer cells. As previously described, NETs have many effects on cancer cells including enhancing their ability to invade into tissue, in addition they can alter their metabolism and subsequently increase their metastatic potential and growth. Several mediators of cancer-NET interaction have been identified including TLR4-NE, tumor-specific integrins, and most recently a surface protein called CCDC25 (35, 56, 63). Targeting these receptors using specific antagonist or knockouts have shown promising results in decreasing tumor cell migration, metastatic niche formation, and growth in murine models (35, 56).

## Conclusion

Liver ischemia and reperfusion injury (IRI) is an important inducer of inflammation. This review discusses the importance of IRI and Neutrophils in mediating tissue damage and transplant rejection. IRI promotes the recruitment of Neutrophils to the liver which can in turn promote transplant rejection and tumor progression of micro metastatic disease. Within the liver Neutrophils/NETs can directly interact with Kupffer cells, macrophages and T cells in the transplanted liver and the TME. Taken together, this review provides an overview of the many roles of Neutrophils and NETs following liver damage *via* IRI.

## Author Contributions

Manuscript preparation: CK, HY, SH, and BP; Manuscript revision: DG and ST; Figure creation CK and HY. All authors contributed to the article and approved the submitted version.

## Conflict of Interest

The authors declare that the research was conducted in the absence of any commercial or financial relationships that could be construed as a potential conflict of interest.

## Publisher’s Note

All claims expressed in this article are solely those of the authors and do not necessarily represent those of their affiliated organizations, or those of the publisher, the editors and the reviewers. Any product that may be evaluated in this article, or claim that may be made by its manufacturer, is not guaranteed or endorsed by the publisher.
